# Supplementary material for: Impact of the COVID-19 Pandemic on Pediatric Emergency Medicine: A Systematic Review
Source: Medicina (Kaunas). 2022 Aug 17;58(8):1112. doi: 10.3390/medicina58081112 (PMC9413323; doi:10.3390/medicina58081112)
Supplement: Supplementary file 1 [file medicina-58-01112-s001.zip › Supplementary file S2.pdf]

Newcastle-Ottawa Scale

Frist Author (year)	Selection<br>＊＊＊＊＊	Comparability<br>＊＊	Outcome<br>＊＊＊
Amidei (2021)	＊＊＊	＊	＊＊
Atti (2020)	＊＊＊＊	＊	＊＊＊
Brisca (2021)	＊	＊	＊＊
Chong (2020)	＊＊＊＊	＊	＊＊＊
DeLaroche (2021)	＊＊＊＊	＊	＊＊＊
Dopfer (2020)	＊＊＊	＊	＊＊＊
Erlichman (2021)	＊＊＊＊	＊	＊＊＊
Finkelstein (2021)	＊＊＊＊	＊	＊＊＊
Goldman (2020)	＊＊＊＊	＊	＊＊＊
Isba (2020)	＊＊＊	＊	＊＊＊
Jang (2021)	＊＊＊＊	＊	＊＊＊
Kruizinga (2021)	＊＊＊	＊	＊＊
Lee (2021)	＊＊＊＊	＊	＊＊＊
Liguoro (2021)	＊＊＊＊	＊	＊＊＊
Matera (2020)	＊＊＊＊	＊	＊＊＊
Matera (2021)	＊＊＊＊	＊	＊＊＊
Pepper (2021)	＊＊＊	＊	＊＊
Pines (2021)	＊＊＊	＊	＊＊
Ramgopal (2021)	＊＊＊＊	＊	＊＊＊
Raucci (2021)	＊＊＊＊	＊	＊＊＊
Shanmugavadivel (2021)	＊＊＊＊	＊	＊＊＊
Shichijo (2021)	＊＊＊＊	＊	＊＊
Silvagni (2021)	＊＊＊＊	＊	＊＊＊
Sokoloff (2021)	＊＊＊＊	＊	＊＊＊
Yamamoto (2021)	＊＊	＊	＊＊
